# Studying APOE2 Protective Effects in Alzheimer's Disease Using Integrative Approaches and a Novel Mouse Model

**DOI:** 10.1002/alz70855_103450

**Published:** 2025-12-23

**Authors:** Zherui Liang, Ayushi Agrawal, Jason Bant, Michela Traglia, Yanxia Hao, Jessica Blumenfeld, David Shostak, Dennis Tabuena, Antara Rao, Oscar Yip, Min Joo Kim, Xueqiao Jiang, Sam De Leon, Claire Ellis, Kevin Wang, Iris Lo, Misha Zilberter, Reuben Thomas, Yadong Huang

**Affiliations:** ^1^ Gladstone Institutes, San Francisco, CA, USA; ^2^ Gladstone institutes, San Francisco, CA, USA; ^3^ Gladstone Institute, San Francisco, CA, USA; ^4^ The J. David Gladstone Institutes, UCSF, San Francisco, CA, USA; ^5^ Gladstone Institute of Neurological Disease, San Francisco, CA, USA; ^6^ Gladstone Institutes and UCSF, San Francisco, CA, USA

## Abstract

**Background:**

Apolipoprotein E (APOE) exists in humans as three major isoforms—APOE2, APOE3, and APOE4. APOE4 has been identified as the strongest genetic risk factor for Alzheimer's disease (AD). On the other hand, APOE2 has been shown to protect against AD relative to APOE3. While APOE4's detrimental effects in AD pathogenesis have been extensively studied, APOE2's protective effects in AD remain unclear and understudied.

**Method:**

To better understand APOE2's roles in AD protection, we generated an isogenic human APOE2 knock‐in (E2) mouse line from the existing human APOE3 knock‐in (E3) mouse line in our lab, then cross‐bred with wildtype human TAU knock‐in (TAU^WT^) mice to generate E2/TAU^WT^ and E3/TAU^WT^ mice. With these mice, we sequentially performed behavioral (Morris water maze), neurophysiological (hippocampal local field potential (LFP) recording), neuropathological (immunohistochemistry), and transcriptomic (single‐nucleus RNA‐sequencing) analysis at 5 and 10 months of age.

**Result:**

Strikingly, we found that the E2/TAU^WT^ mice had significantly improved spatial learning and memory performance than E3/TAU^WT^ mice at 10 months of age, although there was no difference at 5 months of age. The LFP analyses showed that the high‐amplitude sharp‐wave ripple (SWR) abundance and the CA1 SWR‐associated slow gamma power were significantly increased in E2/TAU^WT^ mice, as compared to E3/TAU^WT^ mice, at 10 months of age. Importantly, the improvement of certain LFP signatures in E2/TAU^WT^ mice strongly correlated with their improved spatial learning and memory performance. Furthermore, immunohistochemistry and snRNA‐seq analyses revealed that alterations to certain cell types (or subtypes), such as inhibitory neurons and microglia, were associated with the improved spatial learning, memory performance, and hippocampal network activities.

**Conclusion:**

Taken together, our integrative analyses offer valuable insights into the cellular and molecular mechanisms underlying APOE2's protective roles in AD pathogenesis, providing a strong foundation for developing novel therapeutic strategies that mimics APOE2's protective effects.

